# The Defamatory Article in the Dental Times

**Published:** 1868-08

**Authors:** 


*The Defamatory Article in the Dental Times.*—We have heretofore, carefully avoided publishing anything in the *Am. Journal of Dental Science* which might be regarded as personal; our aim being to speak well of all, and only to censure when we consider such our duty as one of the editors of a Journal devoted to the interests of the dental profession. We intend to pursue the same course in the future without fear or favor, not considering it worth the time which would be necessary to notice such scurrilous attacks as appear in the July Number of the *Dental Times,* over the signature of T. L. B. We do not need a better answer to the remarks which this person has seen fit to make concerning us, than the "Protest" from the Alumni of his own College, published in the July Number of our Journal, against the practice of granting diplomas to men who have not attended lectures, and without a sufficient examination.

In this same defamatory article appears a letter purported to have been written by the youth on whom the faculty of the Pennsylvania Dental College conferred the degree of D. D. S. at their last commencement, on attending *one course* of lectures without having had any previous practice, and concerning whose case the editorial in the May No. of our Journal under the title of "A Degree Easily Obtained," was written.

This letter, which we have no doubt was *doctored* for the occasion, (as the reputed writer, from all the information we have respecting him, does not possess the ability to write even such an one,) accuses us of "acting in an ungentlemanly and unprofessional manner, in my opinion, in a business transaction with a friend of mine."

We feel assured that those who know us best, would require more respectable evidence than T. L. B. or his *protege* could furnish, before believing any such charge. To strangers we will simply say, that if the claim and receipt of the amount of *a note five years due*, against a relative of this youth, be considered "ungentlemanly and unprofessional," then we are guilty of such conduct. As to the refusal of this youth to answer us when addressed, which he deems very important, the truth is, that we only accosted him for the purpose of having him deliver a message to his relative, concerning the note before referred to, and which message we should as soon have sent by a servant boy, had one been present, as by him.

His letter, so far from proving that he had had any practice whatever prior to entering the Pennsylvania College, confirms all that was charged in the editorial of our Journal; indeed, one of the preceptors he refers to is the author of a letter to us protesting against the action of the Pennsylvania College in granting him the degree.

The question naturally arises why, if he had been in practice since 1860 as he alleges, did he not present certificates from the two preceptors instead of the one from a relative, with whom, he says, he remained but a short time? The true reason is that he could not have procured such a certificate from one of these preceptors who is well acquainted with his previous history.

We have at present four students in our office who at times assist us in our practice, yet we have never considered such assistance to amount to actual practice, but as occurring in the course of instruction we give to all students.

The youth also asserts, after making use of a number of epithets very easily written, that the attack upon him originated with us out of revenge to gratify our personal feelings, which is so far from the truth, as to the editorial being any attack upon him, that, had we never beheld him, the same article would have been published as soon as the facts of the case were presented by our correspondents; indeed, the cause he alludes to had been forgotten, or if remembered ascribed to boyish impulsiveness too insignificant for notice.

Having said enough of the youth and his family affairs, we will return to T. L. B. who accuses us of malignant feelings towards the Faculty of the College of which he is the Dean; and on another page is so witty as to bring forward the charge of lunacy.

In answer to the first of these charges we shall simply state that it is an error, and that we shall abstain from the use of any of the epithets so freely bestowed upon us.

As regards the second charge, the July No. of the *Dental Times* is evidence that T. L. B. considers an article from our pen sane enough to reprint among his selected matter. We would also remark that *slang* is no argument.

We made the assertion in the March No. of the *Journal* that we had *good authority* for saying that the Baltimore College had thirty more students than any other, as no less than four reports of the number of students at the other Colleges, received from as many different sources, corresponded, and we did not discover the error until the proceedings of the Commencements were published, which we considered a sufficient correction.

We have, heretofore, been under the impression that five years of actual practice was what the Pennsylvania College professed to consider equivalent to one session, but learn from the article of T. L. B. that four years and eleven months of pupilage and one month of actual practice, or more of pupilage and even less of practice, will be considered as equivalent to one session at this College.

T. L. B. also wishes to make capital, by remarking that a portion of the Faculty of the Baltimore College "were down assisting the rebels" during the war. We have never sought to conceal this fact, and will inform him that no less than four of the present Faculty were so engaged during the time referred to; and if the information will prove of any service to himself and College, we are very willing that they should profit by it. We will also inform him of another fact of which, perhaps, he is ignorant, namely, that *all* the members of the Faculty of the Baltimore College are *natives* of the South; if he imagines that this information will also be of service, he is at liberty to make use of it.

